# Effect of Slow-Release Urea Partial Replacement of Soybean Meal on Lactation Performance, Heat Shock Signal Molecules, and Rumen Fermentation in Heat-Stressed Mid-Lactation Dairy Cows

**DOI:** 10.3390/ani13172771

**Published:** 2023-08-31

**Authors:** Maocheng Jiang, Xuelei Zhang, Kexin Wang, Osmond Datsomor, Xue Li, Miao Lin, Chunyan Feng, Guoqi Zhao, Kang Zhan

**Affiliations:** 1 Institute of Animal Culture Collection and Application, College of Animal Science and Technology, Yangzhou University, Yangzhou 225009, China; jmcheng1993@163.com (M.J.); 15056282672@163.com (X.Z.); awkx5527@163.com (K.W.); datsomorosmond@gmail.com (O.D.); lx929492@163.com (X.L.); gqzhao@yzu.edu.cn (G.Z.); 2 Institutes of Agricultural Science and Technology Development, Yangzhou University, Yangzhou 225009, China; 3 Joint International Research Laboratory of Agriculture and Agri-Product Safety, The Ministry of Education of China, Yangzhou University, Yangzhou 225009, China; 4 Shanghai Menon Animal Nutrition Technology Co., Ltd., Shanghai 200000, China; fengchunyan323@163.com

**Keywords:** slow-release urea, heat shock signal molecules, methane emission

## Abstract

**Simple Summary:**

This study showed that the substitution of 500 g SM with 100 g SRU plus 400 g corn silage led to an improvement in dairy cows’ efficiency due to an enhanced production performance, a lower secretion of heat shock proteins, antioxidant status, and better rumen fermentation. Furthermore, the prediction results indicate that the SRU group exhibits a significant decrease in CH_4_ emissions when producing 1 L of milk compared to the SM group. Thus, future studies should focus on investigating using SRU partially substituted for soybean meal in diets for environmental sustainability to better understand its impact on the production of ruminants and the environment.

**Abstract:**

This study aimed to assess the effects of partially substituting soybean meal in the diet with slow-release urea (SRU) on the lactation performance, heat shock signal molecules, and environmental sustainability of heat-stressed lactating cows in the middle stage of lactation. In this study, 30 healthy Holstein lactating dairy cattle with a similar milk yield of 22.8 ± 3.3 kg, days in milk of 191.14 ± 27.24 days, and 2.2 ± 1.5 parity were selected and randomly allocated into two groups. The constituents of the two treatments were (1) basic diet plus 500 g soybean meal (SM) for the SM group and (2) basic diet plus 100 g slow-release urea and 400 g corn silage for the SRU group. The average temperature humidity index (THI) during the experiment was 84.47, with an average THI of >78 from day 1 to day 28, indicating the cow experienced moderate heat stress conditions. Compared with the SM group, the SRU group showed decreasing body temperature and respiratory rate trends at 20:00 (*p* < 0.1). The substitution of SM with SRU resulted in an increasing trend in milk yield, with a significant increase of 7.36% compared to the SM group (*p* < 0.1). Compared to the SM group, AST, ALT, and γ-GT content levels were significantly increased (*p* < 0.05). Notably, the levels of HSP-70 and HSP-90α were significantly reduced (*p* < 0.05). The SRU group showed significantly increased acetate and isovalerate concentrations compared with the SM group (*p* < 0.05). The prediction results indicate that the SRU group exhibits a significant decrease in methane (CH_4_) emissions when producing 1 L of milk compared to the SM group (*p* < 0.05). In summary, dietary supplementation with SRU tended to increase the milk yield and rumen fermentation and reduce plasma heat shock molecules in mid-lactation, heat-stressed dairy cows. In the hot summer, using SRU instead of some soybean meal in the diet alleviates the heat stress of dairy cows and reduces the production of CH_4_.

## 1. Introduction

The supply and demand of animals by humans rises as people’s living standards increase. Greenhouse effects and global warming have impacted human and animal life activities [1]. Another element that cannot be disregarded in the well-being and productivity of animals is the concomitant heat stress. In fact, farmers are also facing increasing challenges in the process of raising lactating cows, such as improving production efficiency, environmental pollution, greenhouse gas emissions, etc. [2]. Finding strategies to cope with heat stress is the key to sustainable production in dairy cows.

The occurrence of heat stress can affect the production of lactating cows in multiple aspects, such as decreased feed intake and immune function [3]. It is precisely because of these reasons that the lactation performance of cows decreases. More evidence suggests that under heat stress conditions, the yield of cows reduces by 35–40% [4]. When the temperature and humidity index (THI) exceeds 72, cows enter a mild heat stress state, which reduces nutrient metabolism and absorption [5]. Meanwhile, heat stress can induce oxidative stress in cows [4]. In this state, the production of reactive oxygen species exceeds the capacity of the antioxidant defense system, especially in lactating cows, which require a lot of energy to synthesize many milk components [6]. During heat stress, animals exhibit various physiological, endocrine, and behavioral mechanisms to cope with heat stress [7]. Usually, the decrease in feed intake caused by heat stress is considered the main factor leading to a negative energy balance and a reduction in milk production [8]. In addition, the basic physiological mechanisms of the rumen are altered by high environmental temperatures, which have a negative impact on ruminants, leading to an increased risk of health problems and metabolic disorders [9]. It can also reduce rumen motility, affecting feed digestion in the gastrointestinal tract. Research has shown that cows need to consume more energy and protein under heat stress to regulate the effects of heat stress [10]. Therefore, changing the source of protein in the diet and increasing the energy intake of dairy cows may be an effective measure to cope with heat stress.

The best way to increase the protein content in the feed is to choose high-quality soybean meals to provide more protein sources for the body. However, the shortage of protein feed has become a global problem, and global soybean meal prices remain high, increasing feeding costs [11]. Therefore, finding a protein feed to replace soybean meal is an urgent problem that needs to be resolved. Urea, as the most commonly used non-protein nitrogen source, has the advantages of high nitrogen content, low price, and widespread sources. Adding urea to a dairy cow diet is an effective way to alleviate a shortage of protein resources and reduce the feeding cost. However, urea degrades too quickly in the rumen, and the ammonia produced cannot be used effectively and timely by the microorganisms in the rumen; it accumulates in large quantities, which can easily cause ammonia poisoning and limits urea’s application range. In addition, the loss of energy and the reduced efficiency of microbial growth are among the reasons for limiting the use of urea in ruminant diets. The utilization of slow-release technology can maximize the synthesis efficiency of microbial protein; furthermore, it can provide sufficient energy for the body, improve the production performance of animals, and maintain the stability of the rumen environment [12]. Therefore, this study aims to evaluate the impact of partially replacing soybean meal in the diet with slow-release urea (SRU) on the lactation performance, heat shock signal molecules, and rumen fermentation of heat-stressed lactating cows in the middle stage of lactation.

## 2. Materials and Methods

The experiment was conducted at the experimental farm of Yangzhou University (Yangzhou, China), and the Yangzhou University Institution Animal Care and Use Committee (IACUC) approved all procedures involving animals (SYXK (Su) 2016-0019).

### 2.1. Animals, Experimental Design, and Diets

Thirty lactating Holstein cows (days in milking of 191.14 ± 27.24 d, daily milk yield of 22.8 ± 3.3 kg/d, and 2.2 ± 1.5 parity) were randomly assigned to two treatment groups in a completely randomized block design. Within the week before the experiment, the THI and the average ambient temperature were 81.2 and 29.7 °C, respectively. One week before the experiment, the average rectal temperature and respiratory rate were 38.3 °C and 58.4 breaths/min, respectively. The experimental group used 100 g Menogen (slow-release urea) instead of 500 g soybean meal. The basal diet used in this experiment was a total mixed ration (TMR) and concentrate feed diet, and the ingredients and chemical compositions of the feed are shown in Table 1. Menogen was supplied by Shanghai Menon Animal Nutrition Technology Co., Ltd. (Shanghai, China). Cows were placed in separate sheds with free drinking water, fed thrice daily at 105% free feed intake (07:00, 13:00, and 20:00 h), and milked thrice daily at 08:00, 14:30, and 21:00 h. The entire experimental duration was 5 weeks, including a 1-week adaptation period, starting from 20 July 2022.

### 2.2. Environmental Temperature and Humidity

An electronic thermometer and hygrometer (Deli Group Ltd., Shanghai, China) were used to record the ambient temperature and relative humidity in the cowshed area (6 areas in different directions) three times a day (08:00, 14:00, and 20:00 h).

THI values were calculated using the following equation [13]:

THI = [(1.8 × Environment Temperature (°C) + 32] − [0.55 − (0.0055 × Relative humidity)] × [(1.8 × Environment Temperature (°C) − 26]

### 2.3. Rectal Temperature and Respiratory Rate

The rectal temperature and respiratory rate data were recorded using a method described in previous research [3]. The body temperature index (rectal temperature and respiratory rate) was measured thrice daily (08:00, 14:00, and 20:00 h). A mercury-in-glass thermometer was used to measure rectal temperature and to determine the respiratory rate by breathing instances in 1 min.

### 2.4. Sample Collection and Analyses

The dry matter intake (DMI) was determined by recording the amount of feed provided and remaining for three consecutive days each week throughout the experimental period. Feed samples were collected and dried in an oven at 65 °C for 48 h, then ground using a Wiley mill (1188Y, Thomas Willey, Denver, CO, USA) with a 2 mm mesh size, and then stored for further analysis. The feed was then analyzed for dry matter (DM), ash, crude protein (CP), neutral detergent fiber (NDF), acid detergent fiber (ADF), total nitrogen (N), and ether extract (EE).

Digestive and metabolic tests were conducted within 7 days before the end of the experiment, with the first 2 days being the adaptation period and the last 5 days being the sampling period. Feces and urine were collected as described by Jiang [14]. The feces were dried and ground using a grinder (WF-20B, Zhenfeng, China), and then the DM, NDF, ADF, total nitrogen, and ash content were determined. For the TMR and fecal samples, AOAC method 930.15 was used to detect DM; CP was detected via the method described, using a Scino KT260, FOSS, Hillerod, Denmark; NDF and ADF were detected via a fiber optic analyzer (2000i, Ankom, New York, NY, USA) using the method described by Van Soest [15]; total nitrogen was detected via the method described by Santana; and ash content was determined using the AOAC method 942.05 [14].

The collection of milk samples followed the method described by Jiang [14], summarized as mixing the samples collected at three time points every day and storing them with preservatives. Finally, the collected samples were sent to a testing company (Dairy One Cooperative Inc., Shanghai, China) for milk composition analysis. The energy-corrected milk (ECM) value was calculated using the formula of Jiang [14]:

ECM = (0.3246 × kg of milk) + (13.86 × kg of milk fat) + (7.04 × kg of milk protein).

During the entire experimental period, blood was collected three times (at weeks 0, 2, and 4), and blood samples were collected from each individual’s jugular vein before milking. Blood samples were centrifuged (4 °C and 3500 rpm), followed by plasma and serum collection and storage at −80 °C for further analysis. The samples were delivered to Yangzhou Hengyi Biological Company for testing (Yangzhou, China). Analysis of plasma AST, ALT, ALP, γ-GT, and T-AA levels was conducted using an automated biochemical analyzer (BS-240Vet, Mindray, Shenzhen, China). Plasma HSP-70 and HSP-90α levels were analyzed via an ELISA kit (Huaying Biological, Beijing, China). Analysis of plasma SOD, GSH-PX, CAT, TAOC, and MDA levels was conducted using an ELISA kit (Huaying Biology, Beijing, China).

The entire rumen content sampled from four locations in the rumen was filtered through four layers of medical gauze. Subsequently, the pH value (PB-21, Beijing Sartorius Scientific Instrument Co., Ltd., Beijing, China) was immediately measured, and the samples were stored at −20 °C for volatile fatty acid (VFA) and ammonia nitrogen (NH_3_) determination. VFA was determined by gas chromatography (Thermo Fisher Scientific, Waltham, MA, USA) using a detection method based on previous research by Wu et al. [16], and the technique described by Lamminen was used to detect NH_3_ [17].

### 2.5. Environmental Impact: Predicted Enteric Methane Production

According to the experimental method described by Grossi [18], the correlation coefficient between the predicted value and the measured value reached 0.880. Therefore, the prediction was made according to the following formula:

CH_4_ (g/d) = 2.54 + 19.14 × DMI

where:

1. CH_4_ = enteric methane production;
2. DMI = dry matter intake (kg/head/day).

### 2.6. Statistical Analysis

Data are presented as means and the standard error of the mean (SEM). The statistical analysis software package SPSS 20.0 (IBM Corp., Armonk, NY, USA) was used to perform an ANOVA test and the unpaired Student’s *t*-test [14]. *p*-values < 0.05 were considered statistically significant.

## 3. Results

### 3.1. Measurement of THI and Heat Stress

**Figure 1:**
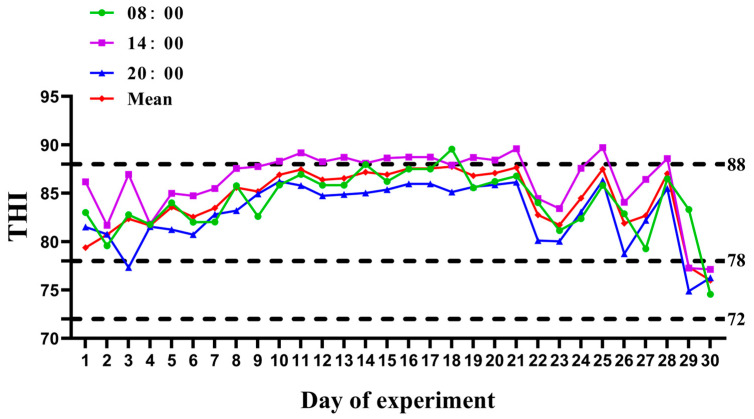
Changes in the THI during the experimental period.

The average daily ambient temperature of the cowshed at 06:00, 14:00, and 20:00 h was 31.31, 34.36, and 30.37 °C, respectively. As shown in Figure 1, the average THI during the experiment was 84.47, with an average THI of >78 from day 1 to day 28 (Figure 1). This indicates that a cow has been in a state of heat stress during the experiment, and most of the time within the range of a moderate heat stress environment.

### 3.2. Rectal Temperature and Respiratory Rate

The body temperature and respiratory rate of cows were monitored at different time points daily. Compared with the control group, the SRU group showed decreasing body temperature and respiratory rate trends at 20:00 (Table 2, *p* < 0.1). During other time periods, there was no notable difference in the body temperatures or respiratory rates of the two groups of cows (Table 2, *p* > 0.05).

### 3.3. Lactation Performance

In the process of evaluating the production performance of lactating cows, it was found that after partially replacing SM with SRU, the milk urea nitrogen content in the SRU group was significantly increased compared with the control group (Table 3, *p* < 0.01).

### 3.4. Antioxidant Status and Heat Shock Signaling Molecules

In order to understand the impact of heat stress on the health of experimental cows, plasma metabolites of lactating cows were analyzed (Table 4). Compared with the control group, the AST, ALT, and γ- GT contents were significantly increased in the SRU group (*p* < 0.05). Interestingly, the levels of HSP-70 and HSP-90α were significantly reduced (*p* < 0.01). The results indicated that SRU partially substituting for SM in the diet had a positive impact on relieving heat stress.

### 3.5. Ruminal Fermentation Characteristics

As shown in Table 5, compared with the SM group, the contents of acetate and isovalerate were significantly increased in the SRU group (*p* < 0.05). Partial substitution of SM in the diet with SRU did not significantly affect the rumen fluid’s pH value or NH_3_ concentration.

### 3.6. Environmental Impact: Predicted Methane (CH_4_) Production

This study aims to predict intestinal CH_4_ production based on dry matter intake and assess the sustainability of dairy products by partially substituting SM with SRU (Table 6). The prediction results indicate that the SRU group exhibits a significant decrease in CH_4_ emissions when producing 1 L of milk compared to the SM group (*p* < 0.05). However, no significant difference was observed in the predicted CH_4_ emissions between the two groups. Significantly, this result is only a predicted value, and the actual situation still needs monitoring during production.

## 4. Discussion

Environmental heat stress during summer can lead to hyperthermia in dairy cows [13]. Throughout our experimental period, we observed that the ambient temperature consistently exceeded 30 °C. The average THI remained above 78, and the rectal temperature and respiration rate of SM dairy cows were recorded as 38.88 °C and 59.91 breaths/min, respectively. These findings indicate that the dairy cows were exposed to heat-stress conditions. Rectal temperature and respiratory rate are the most common sensitive indexes to evaluate heat stress in animals, reflecting the degree of heat stress and the intuitive changes made by animals in response to environmental changes. In this experiment, cows were under severe heat stress and extreme heat stress. Dietary supplementation of SRU was associated with decreasing trends in body temperature and respiratory rate at 20:00 h. It has been reported that ruminants mainly dissipate heat from the respiratory tract and skin surface through breathing and sweating [19]. SRU may cause skin vasodilation and transport heat generated by the body to the body surface through blood circulation for evaporative heat dissipation [20], thus alleviating heat stress in cows, but its mechanism needs further study.

Heat stress can reduce rumen microorganisms’ degradation and reproduction ability by changing rumen tissue morphology, rumen temperature, pH, and osmotic pressure, which is not conducive to nutrient digestion and absorption, and thus reduce the production performance and feed conversion rate of animals [21]. Animal studies demonstrate that replacing SM with SRU can be a strategy to enhance dairy cows’ sustainability due to improved production efficiency [18]. Supplementing ruminant diets with SRU can increase the consistency of the link between rumen energy and protein availability and enhance microbial protein synthesis, thus improving its conversion efficiency into milk [22]. Supplementing SRU in the diet can effectively regulate rumen fermentation, including the number of microbial communities, thereby improving production performance. Kowalski et al. [23] noted significantly increased milk production of cows after partially replacing SM with SRU. Giallongo et al. [24] found that the treatment did not affect milk production and dry matter intake. In this study, after partially replacing SM with SRU, there was a trend of increasing milk production.

Heat stress can effect physiological and metabolic changes in animals, including changes in immune function and increased expression of heat shock proteins [25]. Therefore, detecting plasma metabolites in dairy cows is also one of the ways to judge heat stress in dairy cows. Above all, we investigated how supplementing the diet with SRU affects plasma metabolism in heat-stressed dairy cows. Compared with the SM group, the AST, ALT, and γ-GT contents were significantly increased. Studies suggest that compared with mild-heat-stress and no-heat-stress dairy cows, moderate-heat-stress dairy cows had higher expressions of HSF-90α and HSP70 [3]. Interestingly, the levels of HSP-70 and HSP-90α were significantly reduced in the SRU group of our study. The results indicated that SRU partially substituting for SM in the diet did not affect liver function and had a positive impact on relieving heat stress. Very little work has been conducted to evaluate such changes and the biological role of SRU in heat-stressed dairy cows.

The steady state of the rumen environment is necessary to ensure normal digestion and absorption of nutrients [26]. The purpose of SRU products is to reduce the rumen nitrogen release rate while ensuring that all nitrogen is fully available in the rumen. Research has shown that ruminants can utilize non-protein nitrogen (such as SRU) to accelerate the uptake and utilization of nitrogen sources by microorganisms in the rumen, thereby improving the ability of rumen microorganisms to synthesize proteins [27]. Moreover, microorganisms in the rumen can degrade the protein contained in feed into the NH_3_ required by microorganisms. NH_3_ provides a nitrogen source for the synthesis of microbial proteins and plays a vital role for cellulose- and starch-degrading bacteria [28]. This study found that the contents of acetate and isovalerate were significantly increased in the SRU group compared with the SM group. However, there was no significant change in the concentration of NH_3_ in the rumen fluid. This may be due mainly to the lower proportion of SRU partially substituting SM in this experiment. This study found that supplementing the diet with SRU improved rumen fermentation ability, which is consistent with the research of Yang et al. [13].

Greenhouse gas emissions are one of the causes of global warming, exacerbating heat-stress effects on animals [29]. To address these issues, developing more efficient and sustainable feeding methods or diets for dairy production is necessary. Strategies to directly reduce rumen CH_4_ production and improve overall production efficiency can be considered. Replacing protein sources with non-protein nitrogen instead of SM may be an effective strategy to address these challenges, mainly due to the high impact of SM on the environment and the positive impact of non-protein nitrogen on rumen microorganisms [30], which reduces feed costs and improves the protein utilization rate for dairy cow diets. In recent years, this strategy has received increasing attention for reducing the environmental impact of dairy products and improving the productivity and efficiency of cows. Meanwhile, the prediction results indicate that the SRU group exhibits a significant decrease in CH_4_ emissions when producing 1 L of milk compared to the SM group. Specifically, dietary supplementation with SRU tended to reduce plasma heat shock molecules in mid-lactation, heat-stressed dairy cows. In the hot summer, using SRU instead of some soybean meal in the diet alleviates the heat stress of dairy cows and reduces the production of CH_4_.

## 5. Conclusions

This study showed that substituting 500 g SM with 100 g SRU and 400 g corn silage improved dairy cows’ efficiency due to an enhanced production performance, a lower secretion of heat shock proteins, antioxidant status, and better rumen fermentation. Furthermore, the present study showed that the impact of CH_4_ emissions could be reduced when producing 1 L of milk by including SRU as an alternative protein source. Therefore, future research should investigate the effects of using SRU instead of different proportions of soybean meal in the diet on the production performance and greenhouse gas emissions of heat-stressed lactating cows in order to better understand its impact on ruminant production and the environment.

## Figures and Tables

**Table 1:** Ingredient and chemical composition of the experimental diets.

Items	Soybean Meal Group (SM)	Slow-Release Urea Group (SRU)
Ingredient, % of DM		
Alfalfa hay	8.5	8.5
Oaten hay	6.5	6.5
Corn silage	59.2	59.6
Corn grain	5.3	5.3
Soybean meal	4.0	3.5
Slow-release urea	0	0.1
DDGS	3.7	3.7
Oatmeal	4	4
Rootlet	1.6	1.6
Spray corn husk	2	2
Corn germ meal	3	3
Premix ^1^	2.2	2.2
Total	100	100
Nutrient composition		
DM, %	51.38	51.03
Ash, % of DM	6.98	5.67
Crude protein, % of DM	14.93	15.14
Crude fat, % of DM	2.21	2.37
NDF, % of DM	37.56	37.08
ADF, % of DM	21.15	20.95
Ca, % of DM	0.65	0.68
P, % of DM	0.32	0.35
NE_L_^2^, Mcal/kg of DM	1.57	1.53

^1^ Each kilogram of premix provided 55 mg of Mn as MnSO_4_; 62.5 mg of Zn as ZnSO_4_; 0.55 mg of Se as Na_2_SeO_3_; 75 mg of Fe as Fe_2_(SO_4_)_3_; 32 mg of Cu as CuSO_2_; 1.00 mg of I as KI; 0.55 mg of Se as Na_2_SeO_3_; 0.40 mg of Co as CoCl_2_; 1,100,000 IU of Vitamin A; 270,000 IU of Vitamin D; 200,000 mg of Vitamin E; and 4200 mg of Vitamin K3. ^2^ Estimated based on the NRC (2001).

**Table 2:** Effects of SRU on rectal temperature and respiratory rate in heat-stressed dairy cows.

Item	Treatment ^1^		SEM	*p*-Value
	SM	SRU		
Rectal temperature (°C)				
08:00	38.50	38.42	0.05	0.433
14:00	39.21	39.08	0.06	0.392
20:00	38.92	38.73	0.12	0.083
Average	38.88	38.74	0.08	0.224
Respiration rate (breaths/min)				
08:00	50.06	48.83	1.70	0.581
14:00	70.10	64.77	1.53	0.144
20:00	59.55	54.63	1.03	0.071
Average	59.91	56.08	1.55	0.153

^1^ Treatment: SM = soybean meal group; SRU = slow-release urea group.

**Table 3:** Effects of SRU on lactation performance in heat-stressed dairy cows.

Item	Treatment ^1^		SEM	*p*-Value
	SM	SRU		
DMI, kg/d	22.45	21.47	0.49	0.342
Milk yield, kg/d	23.52	25.25	0.35	0.081
ECM ^2^, kg/d	25.80	27.70	0.52	0.423
Milk fat, %	4.03	4.04	0.08	0.310
Milk protein, %	3.52	3.54	0.11	0.611
Milk lactose, %	5.23	5.21	0.19	0.481
Total solids, %	17.09	16.85	0.63	0.302
SCC, ×10^3^/mL	181.21	217.88	12.58	0.582
MUN ^3^, mg/dL	13.99	15.09	0.25	<0.001

^1^ Treatment: SM= soybean meal group; SRU = slow-release urea group; ^2^ ECM = energy-corrected milk; ^3^ MUN = milk urea nitrogen.

**Table 4:** Effects of SRU on nitrogen utilization in heat-stressed dairy cows.

Item	Treatment ^1^		SEM	*p*-Value
	SM	SRU		
AST, U/L	70.08	96.32	5.08	0.007
ALT, U/L	23.04	28.69	1.11	0.008
ALP, U/L	35.68	32.37	1.96	0.411
γ-GT, U/L	20.60	29.15	1.64	0.006
T-AA, μmol/L	3.50	3.64	0.06	0.234
SOD, U/mL	84.87	83.09	2.27	0.704
GSH-PX, U/mL	350.42	365.10	6.82	0.292
CAT, U/mL	24.04	25.54	1.29	0.573
TAOC, U/mL	5.65	5.91	0.34	0.705
MDA, nmol/mL	4.84	4.59	0.14	0.382
HSP-70, pg/mL	343.11	299.79	9.13	0.014
HSP-90α, pg/mL	49.09	40.86	1.41	0.002

^1^ Treatment: SM = soybean meal group; SRU = slow-release urea group.

**Table 5:** Effects of SRU on ruminal fermentation characteristics in heat-stressed dairy cows.

Item	Treatment ^1^		SEM	*p*-Value
	SM	SRU		
Rumen pH	6.58	6.65	0.06	0.595
TVFA (mM)	93.41	104.86	3.38	0.090
Acetate (mM)	59.38	68.53	2.25	0.034
Propionate (mM)	19.67	21.44	1.00	0.403
Butyrate (mM)	10.94	12.10	0.41	0.161
Isobutyrate (mM)	0.88	0.85	0.05	0.897
Valerate (mM)	1.28	1.14	0.06	0.294
Isovalerate (mM)	1.29	0.79	0.11	0.010
Acetate/Propionate	3.07	3.23	0.09	0.402
NH_3_ (mg/dL)	12.99	17.50	1.55	0.153

^1^ Treatment: SM = soybean meal group; SRU = slow-release urea group.

**Table 6:** Environmental impact: predicted enteric CH_4_ production in the two groups.

Item	Treatment ^1^		SEM	*p*-Value
	SM	SRU		
CH_4_, g/d	432.30	413.56	9.44	0.336
CH_4_, g/L milk	18.38	16.37	0.45	0.022

^1^ Treatment: SM = soybean meal group; SRU = slow-release urea group.

## Data Availability

Not applicable.
